# Septic Shock Due to Clostridium perfringens

**DOI:** 10.7759/cureus.4262

**Published:** 2019-03-18

**Authors:** Raynieri Fernandez, Andrea Anampa-Guzmán

**Affiliations:** 1 Internal Medicine, Advocate Illinois Masonic Medical Center, Chicago, USA; 2 Internal Medicine, Main National University of San Marcos, Lima, PER

**Keywords:** sepsis, infection, shock

## Abstract

Clostridium perfringens is an anaerobic Gram-positive bacterium. We present the case of a 75-year-old female presented to the emergency department with progressively worsening acute on chronic left hip pain. It was not until the third day of hospitalization when the initial blood cultures finalized as Clostridium perfringens, antibiotics were changed to piperacillin-tazobactam and clindamycin. Despite the latest measurements, the patient continued getting hypotensive and progressed to multiorgan failure resulting in death by the end of the third day. The recognition of risk factors in addition to gram-positive rod cultures in the setting of septic shock reinforces the importance of appropriate and early empiric antibiotics management and infectious disease consultation to avoid fatal complications.

## Introduction

Clostridium perfringens is an anaerobic Gram-positive bacterium that can be found in the gastrointestinal tract, female genital tract, and on the skin. Infection with C. perfringens can cause gas gangrene, septic shock, myonecrosis, liver abscess, and hemolysis [1, 2]. Clostridial toxic shock syndrome is a life-threatening, rapidly progressive toxigenic illness that can be caused by infection of Clostridium perfringens [3]. Most cases occur during the post-partum period or following a spontaneous or therapeutic abortion. It can also occur in immunocompromised patients but is rarely found in otherwise healthy people [4]. Advanced age, diabetes mellitus type II, renal insufficiency, and malignancy are well-known risk factors for acquiring Clostridium bacteremia [5]. Due to the high mortality rate, early management is crucial.

## Case presentation

The patient is a 75-year-old female with a past medical history of chronic obstructive pulmonary disease, type 2 diabetes mellitus, atrial fibrillation not on anticoagulation. She had multiple hospital admission due to urinary tract infection and left hip pain due to fracture status post open reduction and internal fixation three months before that presented to the emergency department with acute on chronic left hip pain. Patient’s pain started two weeks ago, and she described it as severe, sharp, constant, radiated to the left thigh, that has been progressively worse to the point that she has been unable to walk; nothing had made the pain better. On exam, she had an externally rotated left lower extremity, tender, cold to palpation, along with a 3-cm decubitus ulcer around the heel.

Initial laboratory workup showed elevated potassium of 7.2, creatinine at 13.34 mg/dL, blood urea nitrogen of 103, initial white blood cell counts 51,200/mcl, platelet 585, lactic acid 7.2, erythrocyte sedimentation rate 97 and C-reactive protein 23. Her electrocardiogram was significant for generalized hyperacute T wave. Given electrolytes abnormalities, a non-tunneled central line catheter was placed for urgent hemodialysis. Before this was able to be done the patient became hypotensive and tachycardic, not responsive to aggressive intravenous fluids resuscitation, requiring initiation of vasopressors. She was taken to the intensive care unit and started on vancomycin and piperacillin-tazobactam for empiric coverage. Additional tests result included a positive urine culture for Escherichia coli along with blood cultures showing Gram-positive rods.

At the time of admission, the primary diagnosis was septic shock secondary to urinary tract infection with suspected blood culture contamination. Further workup included left hip arthroscopy to identify another source of infection due to severe persistent left hip pain and lack of improvement in her overall clinical status, but it was unable to yield synovial fluid. Repeated blood cultures showed no growth for which the urinary tract was suspected to be the primary source of infection. Antibiotics were tailored based on susceptibilities of the urine culture and were changed to Ceftriaxone (Table 1). Nonetheless, her admission was complicated by acute hypoxic respiratory failure requiring mechanical ventilation, as well as the addition of multiple vasopressors.

**Table 1:** Antibiotic resistance of urine culture of Escherichia coli.

	Generic Interpretation	Generic Interpretation Numeric
Ampicillin/Sulbactam	Susceptible	0.016 SUSCEPTIBLE
Cefoxitin	Susceptible	1.0 SUSCEPTIBLE
Clindamycin	Resistant	>=256 RESISTANT
Imipenem	Susceptible	0.19 SUSCEPTIBLE
Metronidazole	Susceptible	0.75 SUSCEPTIBLE

It was not until the third day of hospitalization when the initial blood cultures finalized as Clostridium perfringens (Table 2). Due to the lack of improvement and recent blood cultures results, antibiotics were changed again to piperacillin-tazobactam, and the infectious disease specialist was consulted. Recommendations were made to add clindamycin, considering computed tomography (CT) of the hip/pelvis and lumbar spine. We decided to wait for the final susceptibilities report to discuss surgical exploration of the left hip since it was unable to be determined this as the source of primary infection. Despite the latest measurements, the patient continued getting hypotensive and progressed to multiorgan failure resulting in death by the end of the third day.

**Table 2:** Antibiotic resistance of blood culture of Clostridium perfringens.

	Generic Interpretation	Generic Interpretation Numeric
Ampicillin/Sulbactam	Susceptible	0.016 SUSCEPTIBLE
Cefoxitin	Susceptible	1.0 SUSCEPTIBLE
Clindamycin	Resistant	>=256 RESISTANT
Imipenem	Susceptible	0.19 SUSCEPTIBLE
Metronidazole	Susceptible	0.75 SUSCEPTIBLE

## Discussion

Clostridium species are spore-forming gram-positive anaerobic bacilli ubiquitous soil organisms often found in the digestive tract of humans [6]. C. perfringens produces an alpha toxin, which is associated with cytoskeletal disruption and resulting vascular leak. The early signs of Clostridium toxic shock syndrome are nonspecific; they include lethargy, pallor, nausea, vomiting, and pain at the site of infection. The syndrome is notable for the absence of fever. After 48-72 hours of infection, the patients develop refractory hypotension, severe tachycardia, extensive peripheral edema, effusions, hemoconcentration, and leukemoid reaction. Progression is rapid and death often occurs in hours to days. In this case, the source of infection was not found [6].

The diagnosis is often challenging as fever may be absent and site of infection may be internal. Imaging, such as computed tomography or magnetic resonance imaging, may help to identify sites of infection and expedite surgical debridement. The definitive diagnosis is made by detection of the organism from blood or tissue. As with this case, Gram stain shows Gram-positive club-shaped bacilli. Blood cultures are often negative. Due to the rapid progression of shock and multiple organ failure, death often precedes the diagnosis [7].

The management of this challenging syndrome in the early stages of illness is similar to sepsis, requiring fluid administration, vasopressors, and empiric broad-spectrum antibiotics. Unfortunately, no randomized trials or evidence-based guidelines have defined a treatment regimen for clostridium toxic shock syndrome. The antibiotic regimens directed against Clostridium perfringens used in case series include penicillin four million units IV every four hours plus clindamycin 900 mg IV every eight hours [5,8,9]. Clindamycin, a bacteriostatic antibiotic, is often included in antibiotic regimens due to its ability to suppress toxin production. Emergency surgery may be needed for diagnosis, source control, removal of necrotic tissue, and reducing bacterial toxin burden. The mortality rate can range from 27 to 58% if treated promptly and correctly [10].

## Conclusions

This case represents the fatal progressive course of Clostridium toxic shock syndrome. The diagnosis of Clostridium perfringens in blood cultures is particularly difficult to assess and even can be obscured by other possible pathogens, such as Bacillus, which are often regarded as contaminants, as in this case. The recognition of risk factors in addition to gram-positive rod cultures in the setting of septic shock reinforces the importance of appropriate and early empiric antibiotics management and infectious disease consultation to avoid fatal complications.
